# Reliability test of a smartphone-based measurement tool for the United States general surgical trainees’ intraoperative performance using multivariate generalizability theory: a psychometric study

**DOI:** 10.3352/jeehp.2024.21.26

**Published:** 2024-09-24

**Authors:** Ting Sun, Stella Yun Kim, Brigitte Kristin Smith, Yoon Soo Park

**Affiliations:** 1 Office of Surgical Education, Department of Surgery, University of Utah, Salt Lake City, UT, USA; 2 Department of Educational Leadership, University of North Carolina at Charlotte, Charlotte, NC, USA; 3 Division of Vascular Surgery, Department of Surgery, University of Wisconsin School of Medicine and Public Health, Madison, WI, USA; 4 Department of Medical Education, University of Illinois Chicago, Chicago, IN, USA; Hallym University, Korea

**Keywords:** Internship and residency, Mobile applications, Smartphone, Training support, United States

## Abstract

**Purpose:**

The System for Improving and Measuring Procedure Learning (SIMPL), a smartphone-based operative assessment application, was developed to assess the intraoperative performance of surgical residents. This study aims to examine the reliability of the SIMPL assessment and determine the optimal number of procedures for a reliable assessment.

**Methods:**

In this retrospective observational study, we analyzed data collected between 2015 and 2023 from 4,616 residents across 94 General Surgery Residency programs in the United States that utilized the SIMPL smartphone application. We employed multivariate generalizability theory and initially conducted generalizability studies to estimate the variance components associated with procedures. We then performed decision studies to estimate the reliability coefficient and the minimum number of procedures required for a reproducible assessment.

**Results:**

We estimated that the reliability of the assessment of surgical trainees’ intraoperative autonomy and performance using SIMPL exceeded 0.70. Additionally, the optimal number of procedures required for a reproducible assessment was 10, 17, 15, and 17 for postgraduate year (PGY) 2, PGY 3, PGY 4, and PGY 5, respectively. Notably, the study highlighted that the assessment of residents in their senior years necessitated a larger number of procedures compared to those in their junior years.

**Conclusion:**

The study demonstrated that the SIMPL assessment is reliably effective for evaluating the intraoperative performance of surgical trainees. Adjusting the number of procedures based on the trainees’ training stage enhances the assessment process’s accuracy and effectiveness.

## Graphical abstract

[Fig f3-jeehp-21-26]

## Introduction

### Background/rationale

Formative assessment of surgical trainees’ intraoperative performance is important as it informs a trainee’s readiness for unsupervised practice during a procedure, facilitates the identification of at-risk trainees, and guides appropriate remediation efforts. To that end, the System for Improving and Measuring Procedure Learning (SIMPL), a workplace-based, smartphone-based operative assessment application, was developed to facilitate the assessment of residents’ intraoperative performance and record dictated narrative feedback after each procedure across their graduate surgical training years [1]. Ensuring the reliability of the SIMPL assessment is essential for supporting continuous learning and improving surgical training programs. Reliable assessment data allows for precise tracking of trainees’ progress over time, accurate identification of struggling trainees, timely implementation of educational interventions, and ultimately enhances the overall quality of these programs.

However, SIMPL assessments involve multiple sources of variation in assessment conditions. The procedures being assessed vary in terms of complexity and type. Factors intrinsic to trainees (e.g., postgraduate year [PGY]) can also introduce variability in assessment scores. All these factors pose challenges in consistently applying relevant criteria when assessing the intraoperative performance of trainees. Generalizability theory (G theory) offers a way to estimate the reliability of assessment tools by considering multiple sources of measurement errors and examining their generalizability to other samples or occasions [2]. Through a generalizability analysis, researchers can design effective assessments and determine the optimal number of factors (e.g., procedures) required for reproducible assessments. G theory has been increasingly used in health professionals’ education. A systematic review paper identified 44 studies that employed G theory to examine the reliability of technical skills assessment in medical and surgical education [3]. These studies explored variability in assessments arising from various factors including participants, raters, and cases, in both workplace- and simulation-based assessment settings [3]. Two prior studies have utilized G theory to analyze SIMPL data. Williams et al. [4] employed G theory to determine the minimum number of observations needed for an accurate assessment of operative competency among trainees in General Surgery programs. Another study focused on the number of ratings necessary for a reliable evaluation of procedure difficulty, using a linear mixed model and generalizability analysis [5]. Notably, both studies examined the variances attributed to raters, trainees, and programs, and estimated the minimum number of ratings required for a reproducible assessment. So far, however, no studies have been conducted to examine variability resulting from procedures. Previous literature has shown that procedure-related factors, such as procedure type and case complexity, were significant factors contributing to variability in operative assessment [6,7]. Moreover, since the number, type, and difficulty of procedures assigned to each trainee vary, it is important to consider procedures as a factor that influences measurement precision.

### Objectives

In our study, we adopted a multivariate G theory approach to examine the reliability of the SIMPL assessment and to determine the number of unique procedures necessary for reliable assessments.

## Methods

### Ethics statement

The study was deemed exempt by the University of Utah Institutional Review Board given the nature of the research (IRB #00161772). Informed consent was waived because there was no direct contact with participants, and all participant information was fully de-identified.

### Study design

It is a retrospective observational study with national data collected from September 2015 to June 2023.

### Setting and participants

A total of 4,616 residents and 2,763 faculty surgeons from 94 General Surgery Residency programs that utilized the SIMPL smartphone application provided by the Society for Improving Medical Professional Learning were included in the study. Participation was voluntary, and their information was de-identified for analysis purposes. This study followed the Strengthening the Reporting of Observational Studies in Epidemiology (STROBE) reporting guideline [8].

### Variables

Each SIMPL assessment consists of 3 behaviorally-anchored rating items. The first item evaluates the degree of autonomy granted to residents on a validated 4-level Zwisch scale: “show and tell,” “active help,” “passive help,” and “supervision only” [1]. The second item assesses the resident’s performance (i.e., readiness for unsupervised practice) on a 5-level scale, ranging from “unprepared/critical deficiency,” “unfamiliar with procedure,” “intermediate performance,” “practice ready,” to “exceptional performance.” The desired target goals for training are achieving a level of “passive help” on the autonomy scale and “practice ready” on the performance scale, indicating the ability to perform independently and safely. The third item requires raters to assess the complexity of the operative case on a 3-level scale: easiest 1/3, average 1/3, or hardest 1/3 of cases. While the case complexity item was not used for computing variance components and reliability estimation, it was used to sort data. Cases with similar case complexity were selected for analysis.

### Data sources/measurement

Following each procedure, either the attending or trainee initiates an assessment using the SIMPL app on their mobile device. Subsequently, an automatic notification is sent to the other party to prompt their participation in the assessment. Both parties independently complete the assessment, unaware of each other’s assigned scores. It is important to note that assessments have a time sensitivity, expiring after 72 hours following the procedure, as research suggests that assessments beyond this timeframe may lack reliability [9]. Participants are required to undergo a comprehensive 1-hour rater-training session before utilizing the SIMPL app to ensure the response process is valid for the ratings. Participants receive clear definitions for each rating option associated with the constructs and have the opportunity to practice their ratings by engaging with procedure videos and discussing with peers.

### Bias

Several approaches were taken to address potential sources of bias. Since our data is drawn from a national sample, sampling bias is minimized, which helps mitigate selection bias and ensures a more representative sample. Additionally, we stratified our analysis by PGY and case complexity to account for differences in trainee experience and procedure difficulty, reducing the risk of confounding variables affecting the results.

### Sample size

Following sample size guidelines for conducting a generalizability study (G study), a minimum sample size of 400 subjects is recommended to achieve reliable estimates [10]. Our study meets this recommendation, ensuring a robust sample size even after stratification by PGY and case complexity.

### Statistical methods

Given that each evaluation encompassed items measuring different constructs and involved varying pairs of faculty and trainee raters, we adopted a multivariate generalizability design. Rater and item served as fixed effects, while the procedure was considered as a random facet. We stratified the dataset by PGY and conducted separate G studies for each subset of data. To account for variations in the number and complexity of procedures assigned to trainees, we implemented a sampling strategy to ensure a balanced representation. Specifically, each resident was assigned 3 procedures in the sampled datasets. A sample size threshold for each procedure of 50 of the number of trainees is established based on previous research [11]. The sample size threshold of 50 was chosen to ensure accurate estimation of variance components to draw valid interpretations and inferences about the assessment reliability. After constructing the datasets, we first conducted G studies for each dataset to estimate the variance components associated with procedures at each level across PGY. We then performed decision studies (D studies) and estimated statistics, including universe score variance, absolute error variance, relative error variance, generalizability coefficient, and index of dependability (reliability).

The purpose of the G study is to estimate variance components associated with random effects (in this case, procedures). In contrast, the D study focuses on efficient measurement design for practical and operational use by determining the optimal number of procedures based on the variance estimates from the G study. Typically, a value of 0.8 is deemed acceptable for reliability indices [12]. Additionally, we varied the number of procedures to determine the minimum number of procedures required for a reproducible assessment. Lastly, inter-rater reliability between faculty and trainee was calculated using dis-attenuated correlation (Supplement 1). Data were analyzed using the R package “gtheory” (https://cran.r-project.org/) [13]. A detailed description of the statistical analysis is provided in Supplement 2. R codes for analyses are available in Supplements 3–7.

## Results

### Participants and descriptive statistics

A total of 356,038 evaluations were completed for 4,616 trainees following 2,035 unique procedures. Of these, 53.6% (n=2,473) of the trainees and 67.3% (n=1,859) of the faculty were male. The distribution of participants, evaluations, and procedures based on PGY is displayed in Table 1. As expected, the mean ratings for autonomy and performance show an upward trend across PGY levels. The median rating for autonomy is “active help” for the first 3 PGY years, and “passive help” for the last 2 PGY years. The median rating for performance is “intermediate performance” for the first 3 years and “practice ready” for the final 2 years (Table 2).

### G-study variance component

The primary objective of the present study was to estimate the G study variance component of the procedure at every PGY. An example of the variance and covariance components (PGY 5) is provided in Table 3. Notably, the variance components associated with faculty were consistently smaller than those associated with trainees. It is also worth noting that while autonomy encompasses 4 levels and performance includes 5 levels, the variance components associated with autonomy were not always smaller than those linked to performance. However, within each level, variance components for procedures were consistently smaller, suggesting that the variability attributable to the procedure was not substantial. This trend remained consistent across different PGY training years.

### D-study reliability

The composite D-study statistics are shown in Table 4. The generalizability coefficients ranged from 0.717 (PGY 3) to 0.786 (PGY 4) and the index of dependability coefficients ranged from 0.709 (PGY 3) to 0.774 (PGY 4). Based on the estimated variances, we can determine that a trainee’s observed rating, with an added margin of ±0.14 (PGY2), ±0.15 (PGY3), ±0.16 (PGY4), and ±0.16 (PGY5), constitutes a 95% confidence interval for the trainee’s true rating score.

### Number of procedures

**Fig. 1.**
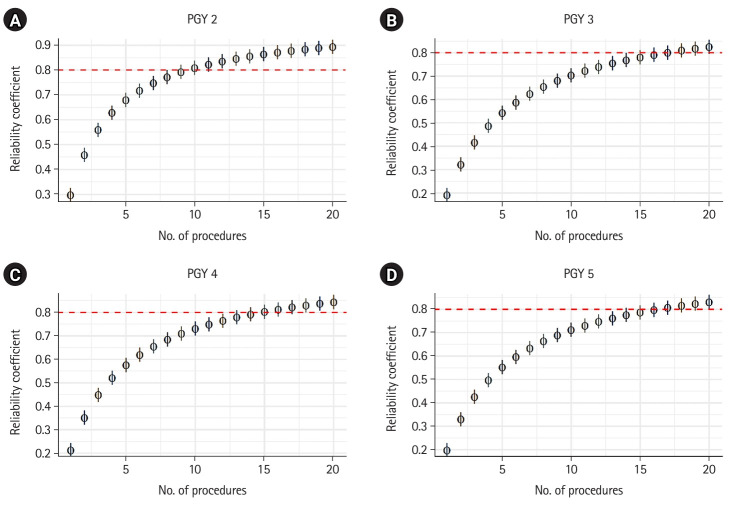
(A–D) The relationship between the reliability coefficient (index of dependability) and the number of procedures. PGY, postgraduate year.

**Fig. 2.**
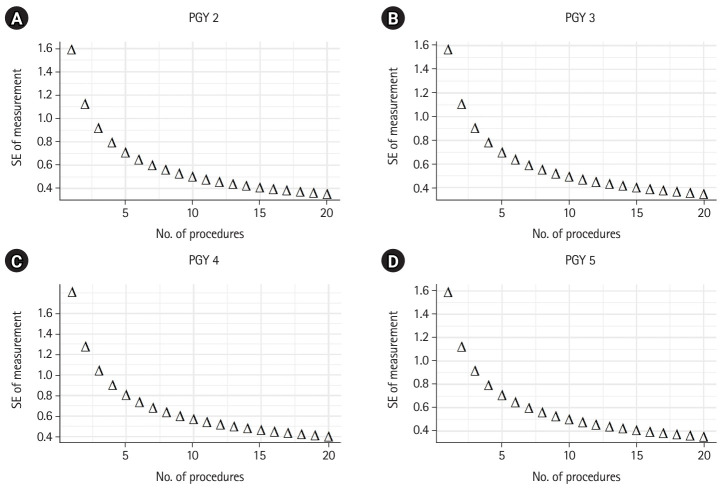
(A–D) The relationship between standard error (SE) (square root of absolute error variance) of measurement and the number of procedures. PGY, postgraduate year.

To determine the minimum number of procedures needed to achieve a reproducible and reliable evaluation of residents’ autonomy and performance at each PGY level, we set a threshold value of 0.8 for the reliability coefficient (i.e., the index of dependability). As illustrated in Fig. 1, we observed an improvement in reliability and a decrease in the standard error of measurement (SEM) as the number of procedures increased, which aligns with our expectations. To attain the index of dependability of 0.8, we found that the optimal numbers of procedures were 10, 17, 15, and 17 for PGY 2, PGY 3, PGY 4, and PGY 5, respectively. Overall, evaluations of residents in the senior years required a larger number of procedures compared to those in the junior years. Fig. 2 provides a visual representation of the relationship between SEM and the number of procedures. It is important to note that although SEM tends to decrease with an increasing number of procedures, the rate of decrease slows down after reaching 7 procedures.

### Inter-rater reliability

The dis-attenuated correlation between faculty and trainees in their assessment of autonomy varied from 0.71 (PGY 2 and PGY 3) to 0.88 (PGY 4), whereas it ranged from 0.45 (PGY 5) to 0.63 (PGY 2) for the assessment of performance. Notably, trainees consistently assigned themselves lower scores in comparison to the scores given by faculty members across PGY levels (Table 5).

## Discussion

### Key results

Medical education is currently undergoing a transition towards competency-based medical education (CBME), an outcomes-based model focusing on learners’ demonstrated knowledge and skill acquisition [14,15]. CBME underscores the importance of reliable assessments to inform decisions concerning a trainee’s competence and, consequently, their preparedness for independent practice [15,16]. The study demonstrated that the SIMPL assessment is reliably effective for evaluating the intraoperative performance of surgical trainees. Adjusting the number of procedures based on the trainees’ stage in training enhances the accuracy and effectiveness of the assessment process.

### Interpretation and comparison with previous studies

Our findings consistently showed modest variability associated with attending faculty compared to trainees, indicating that faculty provided a more reliable assessment of residents’ intraoperative performance compared to trainee self-assessment despite both groups undergoing rigorous rater training. The relatively higher reliability provided by faculty can be attributed to their extensive knowledge and professional experience in the surgical field [17]. These findings suggest that faculty ratings serve as a benchmark against which trainee self-assessment can be compared, which can be used to identify areas where trainees may underestimate or overestimate their performance or autonomy. The finding also highlights the importance of providing additional support to trainees to improve the accuracy of their self-assessments. Such support could include more opportunities for self- or peer observation and evaluation, training sessions on the use of SIMPL assessment tools, and structured feedback on their performance [18].

Our finding revealed that with 3 procedures, the reliability of the assessment of surgical trainees’ intraoperative autonomy and performance exceeded 0.7, which, while not perfect, can be considered satisfactory. This information supports the continued use of SIMPL to evaluate the competence of surgical trainees. We discovered that the optimal number of procedures required for a reproducible assessment was 10, 17, 15, and 17 for PGY 2, PGY 3, PGY 4, and PGY 5, respectively. It is important to note that these numbers differ from the previous study by Williams et al. [4], in which the optimal number of procedures was found to be 60 and 40 for autonomy and performance, respectively. The disparities arise because the study by William et al. [4] aimed to determine the number of ratings (i.e., whether they are the same or from different procedure types), whereas our study aimed to specify the number of unique procedures. Furthermore, our study accounted for variations in PGY and case complexity, rather than using an undifferentiated mix of procedures, averaging across all years of trainees and case complexity.

Despite the requirement of more than 10 procedures for a reliable assessment, an exploration of the data noted that a notable portion of trainees (10.9%) were assessed with only 1 procedure, and more than half (52.0%) were assigned with fewer than 10 unique procedures throughout their 5 years of training. In PGY 5 alone, 10% of trainees had assessments on 1 specific procedure, and 67.4% were assigned fewer than 17 procedures. The current study emphasizes the importance of considering an increased number of procedures for a reliable assessment and provides valuable insights for program directors and faculty.

Our study also found that evaluating residents in the senior years required a larger number of procedures compared to those in the junior years. This observation can be attributed to the fact that junior-level residents exhibit heterogeneity in their intraoperative performance, resulting in considerable variability in assessment and, consequently, requiring a few procedures. In addition, we also assumed that a substantial proportion of junior residents fell below the threshold of “passive help” on the autonomy scale and “practice ready” on the performance scale. Therefore, accurate and consistent classification of trainees does not demand a large number of procedures. However, as residents progressed through PGY, their performance became more homogeneous, centered around the threshold. As a result, a larger number of procedures became necessary to ensure a reliable assessment of their autonomy and performance. The potential challenges the program may face in increasing the number and variety of surgical procedures might include a limited caseload, faculty availability, and resource constraints in workplace-based assessment.

We found that trainees were assigned lower rating scores in both autonomy and performance than faculty assessment, which was partially consistent with previous literature. In a study conducted by Kim et al. [19] involving 619 residents and 457 attendings from 14 general surgery program trainees, trainees consistently scored themselves lower in autonomy compared to faculty members across different PGY levels and case complexity. However, Alameddine et al. [20] found no significant difference between faculty and trainee assessment regarding the level of autonomy demonstrated by trainees during surgical operations, whereas general surgery trainees tended to rate themselves lower in other skill areas such as depth perception, bimanual dexterity, efficacy, tissue handling, and comfort. Our study supports the notion that trainees may underestimate their autonomy and performance, though the inter-rater reliability was acceptable, especially for the assessment of autonomy. It is crucial to implement interventions such as workshops that specifically focus on enhancing trainees’ self-efficacy and awareness of their autonomy and performance to address this issue. These workshops help trainees better understand their capabilities, ultimately fostering professional growth and development.

### Limitations

This paper acknowledges several limitations that should be considered. First, the study was conducted with only a subset of procedures, focusing on cases with similar complexity, which may limit the generalizability of the results to a broad range of case complexity. Future studies should consider including a wider range of cases to assess the replicability of the findings across different levels of complexity, such as hard or easy cases. Second, this paper employed a multivariate design with the fixed rater facet, which limited the opportunity to explore and compare variance components attributable to raters. In this study, we were unable to consider the rater as a random facet due to the requirement of multiple ratings from more than 2 faculty members to estimate the variance component associated with the faculty rating. Lastly, stratifying the data by PGY posed challenges in obtaining a sample size sufficient for PGY 1.

### Conclusions

This study demonstrates that the overall reliability of the SIMPL assessment exceeded 0.70, supporting its continuous and informed use for the formative assessment of surgical trainees. Moreover, our study highlights the importance of adjusting the number of procedures required for evaluating residents based on their specific stages in training. Recognizing the evolving performance patterns and the increasing variability as residents advance through their PGY can enhance the accuracy and effectiveness of the assessment process.

## Figures and Tables

**Figure f3-jeehp-21-26:**
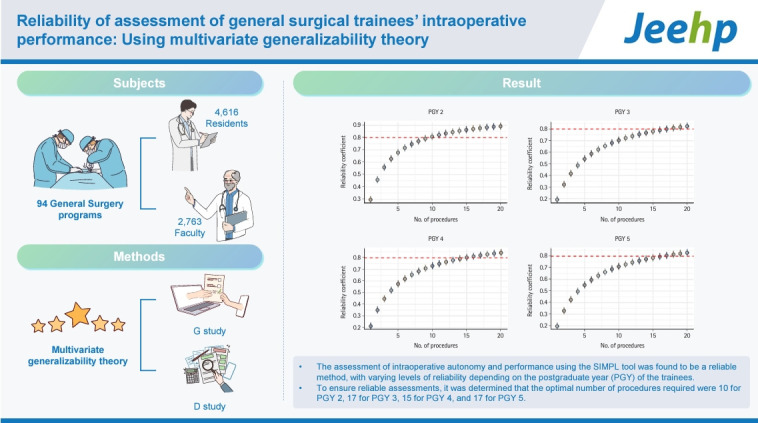


**Table 1.** Demographic characteristics of trainees, faculty attendings, and participating programs

Characteristic	No. (%)
Trainee by gender	
Male	2,473 (53.5)
Female	2,056 (44.5)
Faculty by gender	
Male	1,859 (67.3)
Female	858 (31.1)
Trainee by PGY	
PGY 1	2,170 (47.0)
PGY 2	1,571 (34.0)
PGY 3	1,516 (32.8)
PGY 4	1,404 (30.4)
PGY 5	1,290 (28.0)
No. of programs	94

The total percentage for gender is less than 100% because some residents or faculty did not report their gender. The total number of residents is less than the aggregate across various PGY levels. This discrepancy arises because some residents have had multiple PGY statuses over the years. PGY, postgraduate year.

**Table 2.** Intraoperative autonomy and performance numbers and ratings by training PGY

	PGY 1	PGY 2	PGY 3	PGY 4	PGY 5
No. of assessments by residents	36,749	38,848	45,877	46,650	44,040
No. of assessments by faculty	23,045	25,113	30,979	31,354	33,383
No. of procedures	1,387	1,356	1,368	1,324	1,167
Autonomy					
Mean±SD	1.96±0.75	2.32±0.77	2.54±0.80	2.82±0.80	3.08±0.79
Median (IQR)	2 (1–2)	2 (2–3)	2 (2–3)	3 (2–3)	3 (3–4)
Performance					
Mean±SD	2.77±0.68	3.04±0.65	3.26±0.66	3.55±0.69	3.85±0.63
Median (IQR)	3 (2–3)	3 (3–3)	3 (3–4)	4 (3–4)	4 (4–4)

Zwisch scores are scored as 1 (show and tell), 2 (active help), 3 (passive help), and 4 (supervision only). Performance ratings are scored as 1 (unprepared/critical deficiency), 2 (unfamiliar with procedure), 3 (intermediate performance), 4 (practice ready), and 5 (exceptional performance). PGY, postgraduate year; SD, standard deviation; IQR, interquartile range.

**Table 3.** G study variance and covariance components for PGY 5

	Faculty assessment of autonomy	Trainee assessment of autonomy	Faculty assessment of performance	Trainee assessment of performance
${\hat{\sigma }}^{2}(t)$	0.064 (15.2)	0.090 (12.3)	0.033 (18.5)	0.097 (36.5)
${\hat{\sigma }}^{2}(p)$	0.041 (9.6)	0.084 (4.7)	0.013 (17.3)	0.017 (6.3)
${\hat{\sigma }}^{2}(tp)$	0.317 (75.2)	0.312 (83.0)	0.222 (64.2)	0.152 (57.2)

${\hat{\sigma }}^{2}(t)$, ${\hat{\sigma }}^{2}(p)$, and ${\hat{\sigma }}^{2}(tp)$ refer to variability due to trainees (i.e., universe score variance), procedures, and the interaction between trainee and procedure, respectively. Values in the parenthesis refer to the proportion of variability attributed to each source. PGY, postgraduate year.

**Table 4.** Decision study composite universe score variance, error variance, and reliability coefficients

	PGY 2	PGY 3	PGY 4	PGY 5
Universe score variance	0.068	0.058	0.088	0.061
Absolute error variance	0.020	0.023	0.025	0.024
Relative error variance	0.020	0.022	0.024	0.021
Generalizability coefficient	0.769	0.717	0.786	0.742
Index of dependability	0.765	0.709	0.774	0.716

The universe score variance captures the extent of variation in an individual’s true rating score across the entire population. Absolute error refers to the disparity between the actual rating score and the expected rating score of a person. The relative error indicates the discrepancy between a person’s observed deviation score and the universe’s deviation score. The generalizability coefficient and the index of dependability, ranging from 0 to 1, bear resemblance to reliability coefficients. However, the distinction between the 2 lies in the fact that generalizability represents the reliability of a norm-referenced assessment, whereas the index of dependability pertains to the reliability of a criterion-referenced assessment. PGY, postgraduate year.

**Table 5.** Mean rating scores assigned by faculty and trainee and dis-attenuated correlation between faculty and trainee assessment by PGY

Item	PGY 2		PGY 3		PGY 4		PGY 5	
	Autonomy	Performance	Autonomy	Performance	Autonomy	Performance	Autonomy	Performance
Faculty	2.42	2.99	2.64	3.24	3.05	3.68	3.35	3.98
Trainee	2.36	2.72	2.54	3.00	2.84	3.30	3.16	3.65
Dis-attenuated correlation	0.71	0.63	0.71	0.48	0.88	0.60	0.87	0.45

PGY, postgraduate year.

## Supplementary materials

Supplementary files are available from Harvard Dataverse: https://doi.org/10.7910/DVN/F6OOXV

Supplement 1. Equations for calculating the interrater reliability between faculty and trainee using dis-attenuated correlation.

Supplement 2. A detailed description of the statistical analysis.

Supplement 3. R code for dis-attenuated correlation postgraduate 2 (PGY 2).

Supplement 4. R code used to conduct G and D studies.

Supplement 5. R code used to sample datasets.

Supplement 6. R code to plot for reliability.

Supplement 7. R code for the descriptive statistical analysis.

Supplement 8. Audio recording of the abstract.
