# Supplementary material for: Reliability test of a smartphone-based measurement tool for the United States general surgical trainees’ intraoperative performance using multivariate generalizability theory: a psychometric study
Source: J Educ Eval Health Prof. 2024 Sep 24;21:26. doi: 10.3352/jeehp.2024.21.26 (PMC11959404; doi:10.3352/jeehp.2024.21.26)
Supplement: Supplementary file 1 — Supplement 1. Equations for calculating the interrater reliability between faculty and trainee using dis-attenuated correlation. [file jeehp-21-26-suppl1.docx]

**Supplement 1.** Equations for calculating the interrater reliability between faculty and trainee using dis-attenuated correlation

For the assessment of autonomy, the dis-attenuated correlation is given by: $\frac{r_{fa,ta}}{sqrt\left( r_{fa}*r_{ta} \right)},$

and for the assessment of performance, the dis-attenuated correlation is given by: $\frac{r_{fp,tp}}{sqrt(r_{fp}*r_{tp})}$,

where $r_{fa,ta}$ and $r_{fp,tp}$refer to the correlation between faculty and trainee assessments, and the subscripts $r_{fa}$*,* $r_{ta}$*,* $r_{fp}$*,* $r_{tp}$ refers to the reliability of faculty assessment of autonomy, trainee assessment of autonomy, faculty assessment of performance, and trainee assessment of performance, respectively.
