# Supplementary material for: Reliability test of a smartphone-based measurement tool for the United States general surgical trainees’ intraoperative performance using multivariate generalizability theory: a psychometric study
Source: J Educ Eval Health Prof. 2024 Sep 24;21:26. doi: 10.3352/jeehp.2024.21.26 (PMC11959404; doi:10.3352/jeehp.2024.21.26)
Supplement: Supplementary file 2 — Supplement 2. A detailed description of the statistical analysis. [file jeehp-21-26-suppl2.docx]

**Supplement 2.** A detailed description of the statistical analysis

Given that each evaluation encompassed items measuring different constructs and involved varying pairs of raters, we adopted a multivariate generalizability design. Rater and item served as fixed effects across four levels: assessment of autonomy by faculty, assessment of autonomy by trainee, assessment of performance by faculty, and assessment of performance by trainee. The fixed facet refers to the factor that remains consistent or fixed across different measurement procedures [2]. In other words, if the measurement procedure were repeated, the set of items assessing autonomy and performance would remain the same. We chose not to treat items as a random facet because we do not intend to generalize to a different set of items. Similarly, we did not treat raters as a random facet because each assessment involved a different pair of raters; instead, we calculated interrater reliability as a proxy for rater consistency. Conversely, the procedure was considered as a random facet in this study. In essence, we viewed each measurement procedure to have the fixed items and raters (i.e., self-rater and faculty rater) while having a different set of procedures.

Cases involving multiple residents or attending faculty were excluded from the analysis to maintain clarity and minimize potential confounding factors. Additionally, because residents’ autonomy and performance are strongly associated with their postgraduate year (PGY) level [1], we stratified the dataset by PGY and conducted separate generalizability studies (G studies) for each subset of data. To account for the variation in the number and complexity of procedures assigned to resident trainees, we implemented a sampling strategy to ensure balanced representation. Specifically, each resident was assigned three procedures in the sampled datasets. A sample size threshold for each procedure of 50 of the number of trainees is established based on previous research [11]. However, the easiest 1/3 and hardest 1/3 categories did not meet the minimum sample size threshold of 50. The current study focuses only on the cases of average complexity.

After constructing the datasets, we first conducted G studies for each dataset to estimate the variance components associated with the random facet of procedures at each level across PGY. A composite variance component for each PGY was also calculated. Utilizing the estimated variance components, we performed decision studies and estimated several key statistics, including universe score variance, absolute error variance, relative error variance, generalizability coefficient, and index of dependability. The universe score variance captures the extent of variation in an individual’s true rating score across the entire population. Absolute error refers to the disparity between the actual rating score and the expected rating score of a person (i.e., universe score). By knowing the absolute error variance, we can compute the standard error of measurement and estimate the 95% confidence interval within which a resident’s true rating is likely to fall. Relative error indicates the discrepancy between a person’s observed deviation score and universe deviation score. The generalizability coefficient and the index of dependability, ranging from 0 to 1, bear resemblance to reliability coefficients [2]. However, the distinction between the 2 lies in the fact that generalizability represents the reliability for a norm-referenced assessment whereas the index of dependability pertains to the reliability for a criterion-referenced assessment. Given that SIMPL (System for Improving and Measuring Procedure Learning) has a “passing score” and residents who reach the level of “passive help” on the autonomy scale are considered meaningfully autonomous and those reaching the level of “practice ready” on the performance scale are deemed ready for independent practice, index of dependability, and absolute error variance assume greater significance. Typically, a value of 0.8 is deemed acceptable for both indices [12]. Furthermore, we varied the number of procedures to determine the minimum number of procedures required for a reproducible and reliable assessment. Lastly, inter-rater reliability between faculty and trainee was calculated using dis-attenuated correlation. Data were analyzed using the R package “gtheory” [13].
